# Echocardiogram by apical-subcostal protocol in prone position during invasive mechanical ventilation in cardiovascular intensive care unit

**DOI:** 10.1186/s12947-024-00326-y

**Published:** 2024-06-10

**Authors:** César Del Castillo, Fernando Verdugo, Franco Appiani, Francisca Yáñez, Camila Bontá, Carlos Torres-Herrera, Angela Garcia, Zorba Blázquez-Bermejo, Javier Castrodeza, Daniel Requena, Andreina Rodríguez, Arquimedes Silvio, Agustín Gatica, Arnulfo Begazo, Mario Alfaro

**Affiliations:** 1 Cardiovascular department, Hospital DIPRECA, Santiago, Chile; 2 Cardiovascular department, Hospital Clínico San Borja Arriarán, Santiago, Chile; 3 Cardiovascular department, Hospital Militar, Santiago, Chile; 4 Intensive Care Unit, Hospital DIPRECA, Santiago, Chile; 5 Cardiovascular department, Hospital General Universitario Gregorio Marañón, Madrid, Spain; 6 Postgraduate Department, Faculty of Medicine, Universidad Diego Portales, Santiago, Chile

## Abstract

**Aims:**

To evaluate the feasibility of a transthoracic echocardiogram using an apical-subcostal protocol in invasive mechanical ventilation (IMV) and prone position.

**Methods:**

Prospective study of adults who required a prone position during IMV. A pillow was placed only under the left hemithorax in the prone position to elevate and ease the apical and subcostal windows. A critical care cardiologist (prone group) acquired and evaluated the images using the apical-subcostal protocol. Besides, we used ambulatory echocardiograms performed as a comparative group (supine group).

**Results:**

86 patients were included, 43 in the prone and 43 in the supine. In the prone group, the indication to perform an echocardiogram was hemodynamic monitoring. All patients were ventilated with protective parameters, and the mean end-expiratory pressure was 10.6 cmH2O. The protocol was performed entirely in 42 of 43 patients in the prone group because one patient did not have any acoustic window. In the 43 patients in the prone group analyzed and compared to the supine group, global biventricular function was assessed in 97.7% (*p* = 1.0), severe heart valve disease in 88.4% (*p* = 0.055), ruled out of the presence of pulmonary hypertension in 76.7% (*p* = 0.80), pericardial effusion in 93% (*p* = 0.12), and volume status by inferior vena cava in 93% (*p* = 0.48). Comparing prone versus supine position, a statistical difference was found when evaluating the left ventricle apical 2-chamber view (65.1 versus 100%, *p* < 0.01) and its segmental function (53.4 versus 100%, *p* < 0.01).

**Conclusion:**

The echocardiogram using an apical-subcostal protocol is feasible in patients in the IMV and prone position.

**Graphical Abstract:**

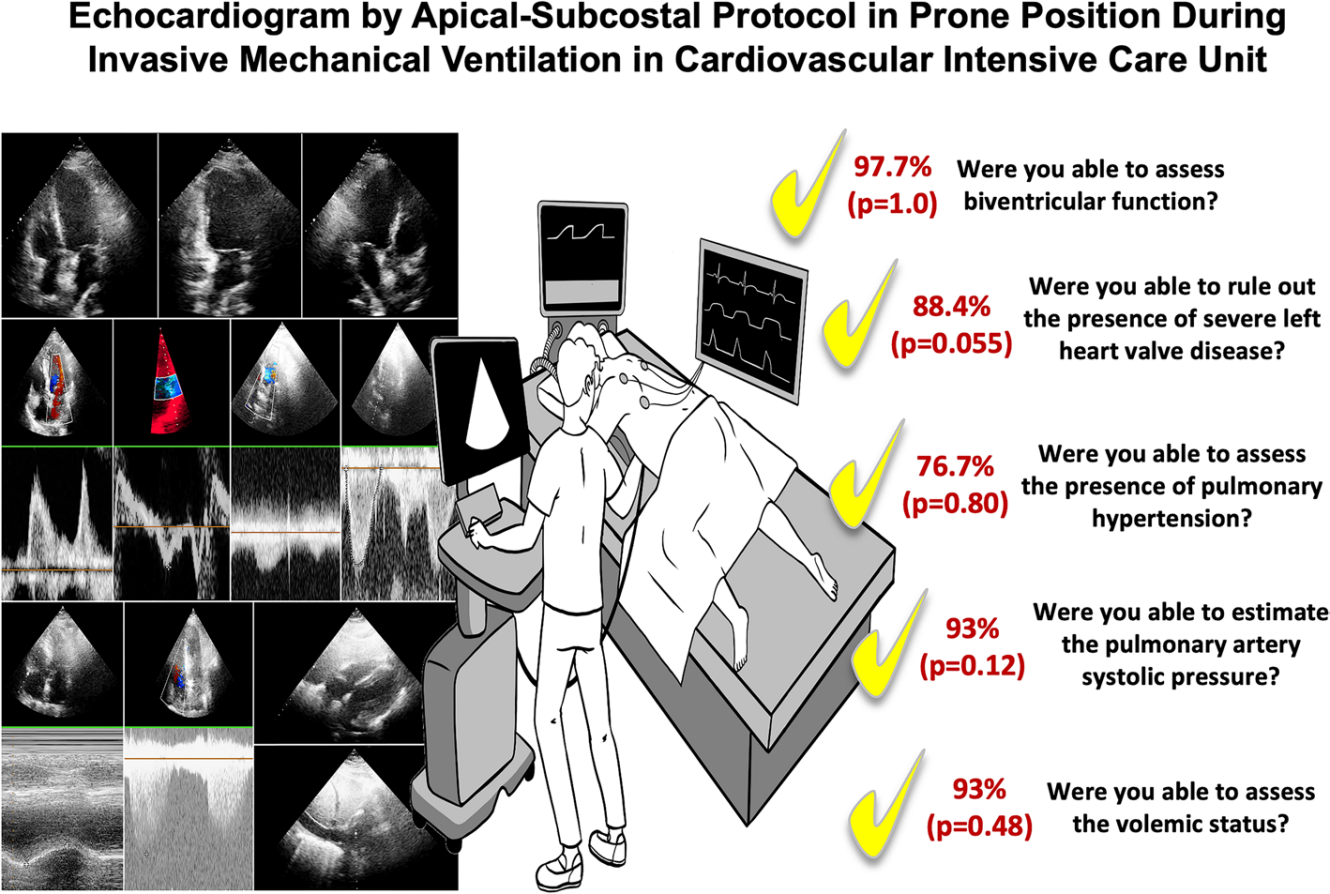

## Introduction

Hemodynamic monitoring is essential in cardiovascular intensive care units (CICU). One diagnostic tool for monitoring is the transthoracic echocardiogram (TTE), which has the advantage of being noninvasive and without adverse events. Biventricular function, heart valvular disease, pulmonary hypertension, pericardial effusion, and volume status can be adequately evaluated [1]. Therefore, we can determine what is affecting the patient's hemodynamic condition.

However, the disadvantage is that the point-of-care echocardiogram must be performed for training people [1]. In the CICU, people are used to having a cardiologist on shift who is prepared to carry out a good-quality echocardiogram. A recent report showed the importance of having a trained physician in the CICU, depicting the prognostic value of echocardiograms in the CICU with some measurements [2, 3].

The invasive mechanical ventilation (IMV) and prone position make it difficult to assess patients’ hemodynamics accurately. Even in expert hands, adequate images or a complete interpretation could be challenging [4]. However, the coronavirus (COVID-19) pandemic has generated the necessity to seek methods for monitoring these patients [4–7].

Consequently, published research regarding echocardiograms in the prone position during IMV has increased. These published protocols are based on an apical window in 4- or 5-chamber, and a few registers include the inferior vena cava by right lateral view [2, 5, 6]. It was feasible to be obtained in a high proportion of patients, greater than 80%, in whom there was possible 4- and 5-chamber biventricular function, LV outflow tract (LVOT) velocity–time integral (VTI) [4, 6, 7], and volume status by measurements of inferior vena cava [6]. However, these studies were small in scale and did not include neither assessment of heart valve disease nor pericardial effusion.

As mentioned before, we decided to investigate the feasibility of the TTE using the “apical-subcostal protocol¨ in a prone position during invasive mechanical ventilation in the cardiovascular intensive care unit.

## Materials and methods

A TTE protocol was performed in consecutive patients older than 15 years who were admitted to the CICU between August 2020 and June 2021. The study was prospective, conducted in three hospitals, and used two different echocardiogram models: Phillips Affinity 50 and General Electric Vivid T9.

Echocardiograms were performed by a cardiologist with university and in-hospital training, experience in cardiovascular critical care for at least two years, and hemodynamic monitoring by echocardiography.

## Protocol

### Patient position (see Fig. 1)

**Fig. 1:**
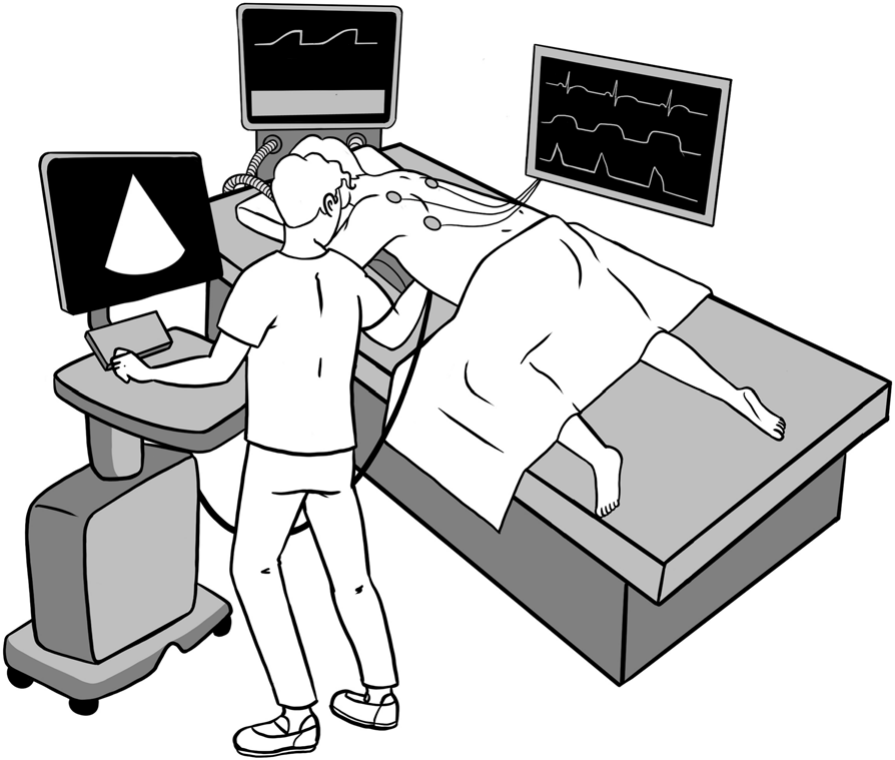
Position to perform apical-subcostal protocol. The head was turned to the left side, the left arm was extended overhead, and the left hip and knee flexed. A pillow was placed only under his left hemithorax to elevate and facilitate an apical and subcostal view of the area. The operator is standing on the left side of the patient and taking the transducer with the right hand. Illustrated by Camila Bonta, MD

During mechanical ventilation and prone position, the patient’s head was turned to the left side, the left arm was extended overhead, and the left hip and knee were flexed. A pillow was placed only under the left hemithorax to elevate and facilitate an apical and subcostal view of the scanning area [8].

#### Operator position and acquisition of the TTE images

The operator stood on the patient's left side, taking the transducer into the right hand. The evaluation began with the apical window, visualizing 4, 5, 2, and 3 chambers.

In the 4-chamber view, the operator performed a visual assessment of the global and segmentary function of the left ventricle (LV), mitral inflow by pulsed-wave Doppler (evaluating E and A wave), pulsed tissue Doppler of the lateral mitral valve annulus (evaluating e’ and s’ wave) and a color-Doppler on the mitral valve looking for any degree of regurgitation. In addition, the basal diameter of the right ventricle (RV), Tricuspid Annular Plane Systolic Excursion (TAPSE), and a continuous-wave Doppler at the tricuspid valve were measured.

In the 5-chamber view, we measured the aortic valve gradient by continuous-wave Doppler, the VTI LVOT by pulsed-wave Doppler, and placed a color-Doppler on the aortic valve to look for some degree of dysfunction. In 2- and 3-chamber views, a visual assessment of global and segmental function, and an evaluation of the mitral and aortic valves were performed.

Subsequently, the operator placed the transducer in the space arranged between the patient and the bed, thanks to the previously placed pillow. In the first instance, a subcostal window of the cardiac chambers is performed to rule out the presence of pericardial effusion and to assess the RV. In addition, the tricuspid regurgitation is sought to evaluate its gradient by continuous-wave Doppler. The transducer was rotated counterclockwise to visualize the inferior vena cava.

#### Images Evaluation

**Fig. 2:**
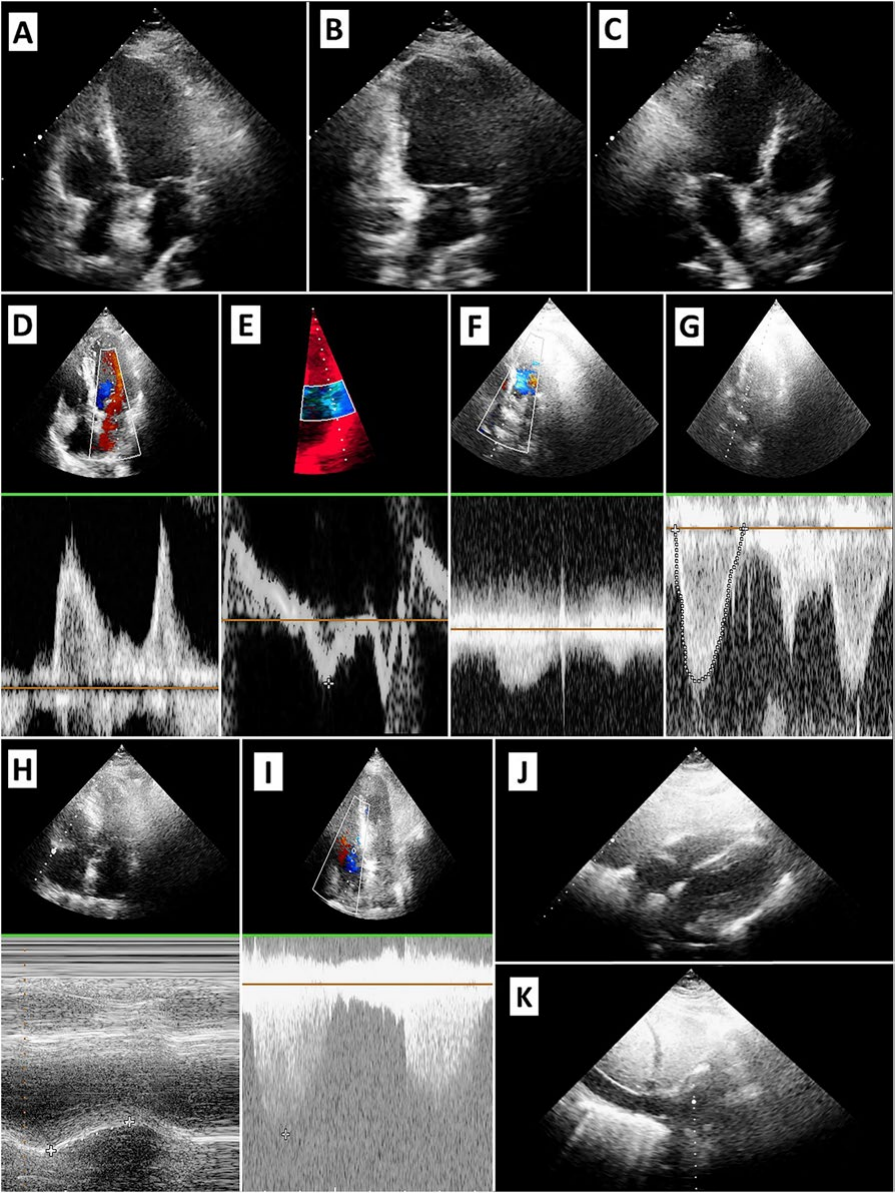
Showing the images obtained by apical-subcostal protocol. A: Apical Four-chamber. B: Apical Two-chamber. C: Apical three-chamber. D: Mitral Inflow by Pulsed-wave Doppler (E and A wave). E: Pulsed Tissue Doppler of the Lateral Mitral Valve Annulus. F: Aortic Flow by Continuous Doppler. G: LV Outflow Tract VTI. H: TAPSE. I: Gradient Insufficient Tricuspid. J: Subcostal Cardiac Chamber. K: Inferior Vena Cava

First, the images were assessed as follows (Fig. 2):

1. Apical window of the 4-, 5-, 3-, and 2-chamber: The valves and LVOT VTI were evaluated using Doppler. The biventricular function, LV segmental function, and TAPSE were also assessed.
2. Subcostal cardiac chambers: The biventricular and valve functions were complemented with apical images. In addition, pericardial effusion and the size of the inferior vena cava were examined.

Second, the images were analyzed in accordance between two cardiologists, who were experts in cardiovascular critical care and echocardiogram monitoring. Both cardiologists obtained a single answer, which could be yes or no.

Third, a set of interpretability questions were formulated, which were answered dichotomously (yes or no) by the two same cardiologist experts in cardiovascular critical care:

- Were you able to assess biventricular function?
- Were you able to rule out the presence of severe heart valve disease?
- Were you able to estimate the pulmonary artery systolic pressure?
- Were you able to assess the presence of pericardial effusion?
- Were you able to determine the presence of the volume state using IVC?

The images and answers obtained were compared with a randomized ambulatory echocardiogram group performed in the echocardiography laboratory by a senior cardiologist (supine group).

Finally, a concordance analysis of the interpretability questions was carried out. The comparison was the same group of two cardiologist experts in cardiovascular critical care with another group of two cardiologist experts in the echocardiogram.

### Ethical aspects

The study protocol was organized to ensure compliance with the Declaration of Helsinki. The Institutional Review Board of DIPRECA Hospital approved the original study protocol as the principal institution (approval certificate number 11, 2022).

### Statistical analysis

A descriptive analysis was conducted on the main relevant demographic and clinical endpoints, as well as a comparative analysis between two groups: The Prone and Control groups. Quantitative variables were expressed as mean ± standard deviation (SD). The chi-square test or Fisher's exact test was used to compare qualitative variables, and the Student’s test or Mann–Whitney U test, when appropriate, was used to compare quantitative variables. Concordance analysis was carried out with the Kappa-value, considering a kappa value between 0.4 and 0.6 indicates moderate agreement, between 0.6 and 0.8 good agreement, and above 0.8 very good agreement. A *p*-value of < 0.05 was considered statistically significant. The data were analyzed using SPSS v29.0.1.0.

## Results

There were 86 echocardiograms performed, 43 patients in the prone group in three different CICUs, and 43 patients in the supine group. The mean age was 52.84 ± 8.88 years, and 62.8% (*n* = 54) were men. Obesity, defined as body mass index > 30 kg/m2, was present in 53.5% (*n* = 46) of the total. The clinical characteristics of both groups are shown in Table 1.

**Table 1:** Basal characteristic. Percentages calculated based on the total of subjects (*n* = 86)

Variable	Prone group *n* = 43	Supine group^a^ *n* = 43	*p*-value
Ager (± DE)	51.07 ± 9.9	54.6 ± 7.35	0.06
Men, %(n)	30.2% (26)	32.6% (28)	0.65
Comorbidities, %(n)			
Hypertension	23.3% (20)	29.1% (25)	0.28
Diabetes	23.3% (20)	25.6% (22)	0.66
Dyslipidemia	18.6% (16)	23.3% (20)	0.38
Tobacco	12.8% (11)	20.9% (18)	0.11
Obesity	30.2% (26)	23.3% (20)	0.19
Invasive mechanical ventilation, n	43	NA	-
Indication for invasive mechanical ventilation, n			
Severe respiratory insufficiency	42	NA	-
Cardiogenic shock	1	NA	-
Tidal volume < 6 ml/kg, yes	43	NA	-
Plateau pressure < 30 mmHg, yes	43	NA	-
PEEP level cmH2O (± DE)	10.6 ± 1.65	NA	-

*NA *Non-applicable

^a^The supine group consisted of patients from the echocardiogram laboratory who attended to evaluate the heart´s function and structure

In the prone-group, the reason to remain in IMV and the prone position was due to severe respiratory failure secondary to COVID-19 infection, except for one patient with cardiogenic shock related to acute myocardial infarction and complicated with severe pneumonia. Two patients analyzed were in vena-venous extracorporeal membrane oxygenation support, both with femoro-jugular cannulation. The indication to perform an echocardiogram was hemodynamic monitoring in 100% of the cases. No complications were associated with performing the protocol. All patients had protective parameters in the mechanical ventilator, with a maximum tidal volume of 6 ml/kg of ideal weight and a plateau pressure of less than 30 cmH2O. The mean end-expiratory pressure (PEEP) was 10.6 ± 1.65 cmH2O. And a total of 60.5% (*n* = 26) were obese.

The protocol in the prone group was successfully performed in 42 out of 43 patients because one had no acoustic window. The feasibility analysis is shown in Tables 2 and 3, and the echocardiographic images are depicted in Fig. 1.

**Table 2:** Show feasibility in obtaining different views and measurements

View and measurement	Prone group *n* = 43	Supine groupa *n* = 43	*p*-value
LV apical 4-Chamber view	97.7% (42)	100% (43)	1.0
LV apical 2-Chamber view	65.1% (28)	100% (43)	< 0.01
LV apical 3-Chamber view	88.4% (38)	100% (43)	0.055
Mitral Inflow by Pulsed-wave Doppler (E and A wave)	97.7% (42)	100% (43)	1.0
Pulsed Tissue Doppler of the Lateral Mitral Valve Annulus (e´and s´wave)	95.3% (41)	100% (43)	0.49
Aortic Flow by Continuous Doppler	97.7% (42)	100% (43)	1.0
LV Outflow Tract VTI	97.7% (42)	100% (43)	1.0
LV Global Function	97.7% (42)	100% (43)	1.0
LV Regional Function	53.4% (23)	100% (43)	< 0.01
TAPSE	95.3% (41)	100% (43)	0.49
Gradient Insufficient Tricuspid	81.4% (35)	83.7% (36)	0.78
Subcostal Cardiac Chamber	86% (37)	93% (40)	0.48
Presence of Pericardial Effusion	93% (40)	100% (43)	0.24
Measure of Inferior Vena Cava	93% (40)	86% (37)	0.48

^a^The supine group consisted of patients from the echocardiogram laboratory who attended to evaluate the heart´s function and structure

**Table 3:** Show feasibility to interpreter and get information by apical-subcostal protocol

Interpretability Questions	Prone group *n* = 43	Supine group^a^ *n* = 43	*p*-value
Were you able to assess biventricular function?	97.7% (42)	100% (43)	1.0
Were you able to rule out the presence of severe left heart valve disease?	88.4% (38)	100% (43)	0.055
Were you able to estimate the pulmonary artery systolic pressure?	76.7% (33)	74.4% (32)	0.80
Were you able to assess the presence of pericardial effusion?	93% (40)	100% (43)	0.12
Were you able to assess the volemic status?	93% (40)	86% (37)	0.48

^a^The supine group consisted of patients from the echocardiogram laboratory who attended to evaluate the heart´s function and structure

Regarding the apical window, a satisfactory apical 4-chamber view was obtained in 97.7%, a 2-chamber view in 65.1%, a 3-chamber view in 88.4%, mitral inflow by pulsed-wave Doppler in 97.7%, and pulsed tissue Doppler of the lateral mitral valve annulus in 95.3%. Aortic flow by continuous-wave Doppler and pulsed-wave Doppler of LVOT were both obtained in 97.7%, evaluating the aortic gradient and VTI LVOT. The LV global function was obtained in 97.7%, while the LV regional function was assessed in 53.4% of the patients. TAPSE measurement was performed in 97.7%, and gradient insufficient tricuspid by continuous-wave Doppler was enabled in 83.7%. The evaluation of aortic, mitral, and tricuspid valves by color-Doppler was assessed in 97.7% of the patients to get an adequate alignment and discard severe valvular heart disease, which was added to the abovementioned measurements. Compared with the supine group, a statistical difference was found when evaluating the left ventricle apical 2-chamber view (*p* < 0.01) and its segmental function (*p* < 0.01).

Subsequently, in the subcostal window, the cardiac chamber view was obtained at 86%, the presence of pericardial effusion was assessed at 93%, and the inferior vena cava was sized up at 93%. There were no differences compared with the supine group (Table 2).

The interpretability questions informed about global biventricular function in 97.7%, ruled out the presence of severe heart valve disease in 88.4%, and estimation of the pulmonary artery systolic pressure in 76.7%, pericardial effusion in 93%, and volume state by IVC in also 93%. No differences were found between both groups (Table 3).

Finally, we assessed the agreement between the interpretability questions against a group of cardiologist experts in the echocardiogram. The kappa value was 0.65 for biventricular function, 0.41 for severe heart valve disease ruling out, 0.47 for pulmonary hypertension assessment, 0.71 for evaluation of pericardial effusion, and 0.67 for volume status.

## Discussion

The present study aimed to analyze an echocardiography protocol in the prone position with the aid of a pillow in the left hemithorax to make the scanning area more accessible. Our main finding shows that an evaluation with transthoracic echocardiography by the apical-subcostal protocol is feasible during IMV in the prone position, even in a group with a high frequency of obesity and moderate PEEP level. Moreover, our protocol has the advantage of allowing a complete assessment through the exact position of the operator (on the left side of the patient).

Besides, this protocol evaluated biventricular function, severe heart valve disease, pulmonary hypertension, pericardial effusion, and blood volume status in most cases. Likewise, the protocol is useful for determining a cardiac cause of hemodynamic deterioration, such as myocardiopathy, the presence of severe heart valve disease, acute pulmonary hypertension, or cardiac tamponade, among other conditions.

**Fig. 3:**
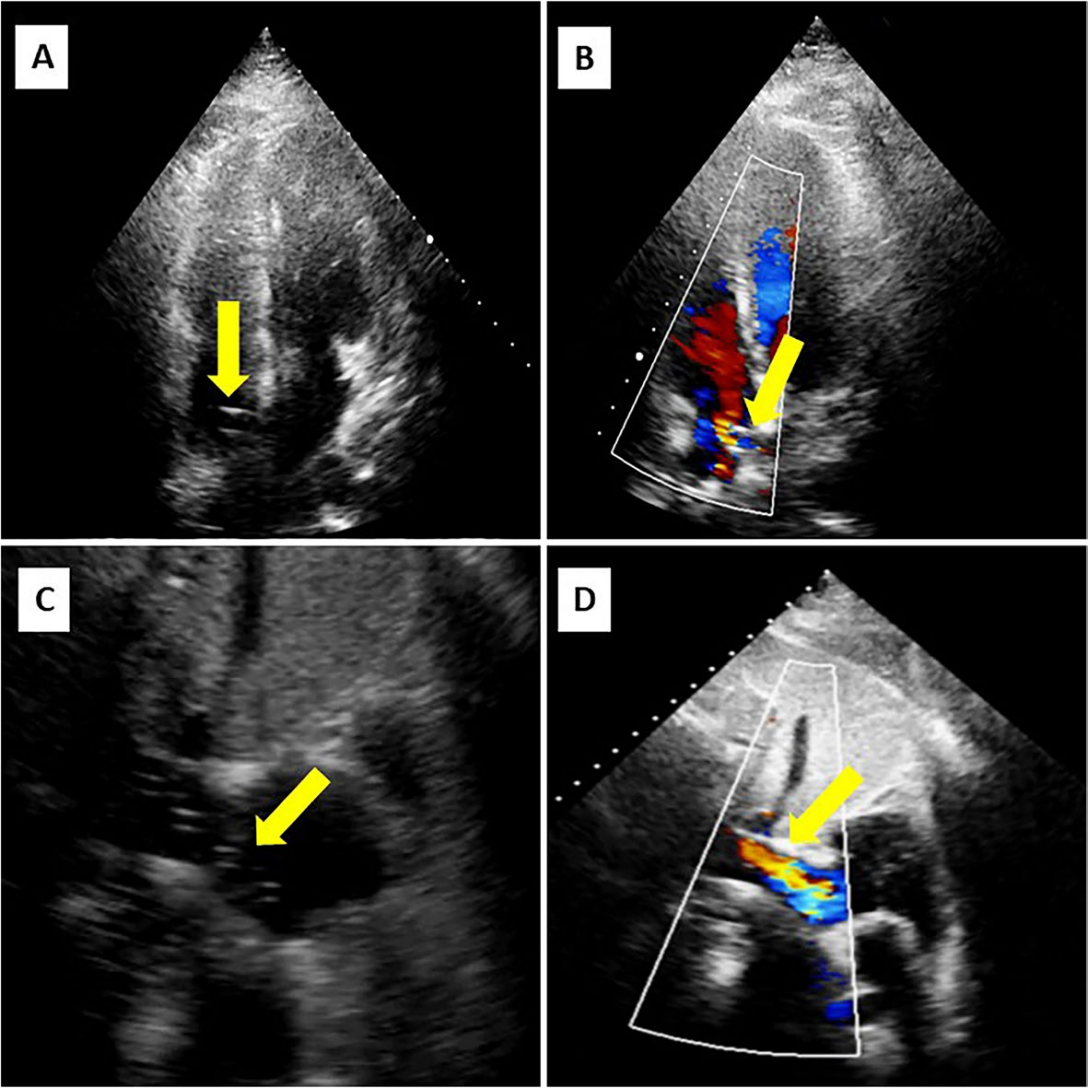
Showing the images obtained by apical-subcostal protocol in Venovenous ECMO patients. Yellow arrows indicate the cannulas and its flow

This protocol has also incorporated two patients in the IMV and prone position under veno-venous extracorporeal membrane oxygenation support (Fig. 3). In both cases, the protocol was performed successfully and without any complications.

Consequently, our research shows how to perform a correct cardiac assessment with echocardiography using an apical-subcostal protocol in different scenarios. We believe that the pillow under the left hemithorax helps improve the mobility of the transducer and to obtain better images, either by apical or subcostal view. In addition to what we hereby describe, it could help that the heart moves approximately 2 cm toward the chest wall in the prone position, and there is a decrease in the interposed lung volume from 15 mm in the supine position to 3 mm in the prone position [9].

Complementing our findings, a high correlation between echocardiogram measurements obtained in the prone and supine positions has been published [4, 5]. We obtained a high assessment rate for the LV apical 4-chamber, whereas there was a low rate for LV 2 and 3-chamber compared with the supine group. However, the LV apical 4-chamber, and its tilt to obtain a 5-chamber, is sufficient to obtain an adequate cardiac assessment [10]. VTI LVOT was achieved in a large number of patients, which is helpful in estimating the cardiac output, adjusting inotropic treatment, and assessing the fluid responsiveness [11]. Similarly, other previous research has published similar results regarding VTI LVOT in the prone position, describing more than 90% of achievement [4, 6, 7].

Furthermore, it would be possible to estimate the left-side filling pressure using the E/e´ ratio or to calculate the pulmonary capillary wedge pressure using different formulas [12], as well as the right-side filling pressure using the inferior vena cava measurement [13]. Recently, it has also been reported that VTI LVOT < 16 cm and E/e’ ratio > 14 were strongly associated with mortality in CICU [14]. Meanwhile, RV function was evaluated successfully in more than 90% of cases, as in other publications [4, 6, 7]. Yet unlike other studies, we have achieved a high percentage of pulmonary hypertension assessment [4] and added the pericardial effusion and heart valve disease evaluation with good results.

Our limitations included that we did not perform pulmonary ultrasound and venous Doppler at the level of the hepatic, portal, and renal veins [15–17], which are fundamental daily management and prognosis of critical patients [17, 18]. We did not also perform invasive hemodynamics monitoring with a pulmonary artery catheter due to respiratory compromise, which could be compared with our echocardiographic measurement. The pulmonary valve and right ventricular outflow tract were not scanned using this protocol, which had been published previously by subcostal view in the prone position [19]. Contrast echocardiography could also enhance the evaluation of the left ventricle [20], particularly LV regional function. Aorta evaluation in the prone position has yet to be described. Otherwise, the variability analysis between the CICU cardiologist and echocardiogram cardiologist was good for biventricular function, pericardial effusion, and volume status. The agreement of pulmonary hypertension assessment showed moderate correlation, but other measurements in the prone position could enhance its evaluation, such as Pulmonary Acceleration Time, Doppler Notch at Pulmonary Valve, and/or End Diastolic Pulmonary Regurgitation Velocity [19, 21]. All these aspects should be incorporated into future protocols.

The clinical implications of our study focus on echocardiography as an available tool to assess the hemodynamic condition of critically ill patients despite the IMV and prone position [22]. It can be complemented with other clinical monitoring elements (lactate and other biomarkers) [23, 24]. The feasibility of this protocol also provides enough time to consolidate the ventilatory treatment through the prone position and subsequently perform a complete cardiac evaluation by echocardiography in the supine position.

## Conclusion

The echocardiogram using the apical-subcostal protocol is feasible in patients who are being assisted by IMV and are in a prone position. Thus, it is useful to evaluate our patients' hemodynamic profiles and conditions.

## Data Availability

The data that support the findings of this study are available on request from the corresponding author, [C.D].
